# Recent advances in extracellular matrix manipulation for kidney organoid research

**DOI:** 10.3389/fphar.2024.1472361

**Published:** 2024-11-06

**Authors:** Ren Wang, Yufei Sui, Qiuyan Liu, Yucui Xiong, Shanshan Li, Wu Guo, Yiwei Xu, Sheng Zhang

**Affiliations:** ^1^ Guangzhou Institute of Cancer Research, The Affiliated Cancer Hospital, Guangzhou Medical University, Guangzhou, Guangdong, China; ^2^ Guangzhou Institutes of Biomedicine and Health, Chinese Academy of Sciences, Guangzhou, Guangdong, China; ^3^ GMU-GIBH Joint School of Life Sciences, Guangzhou Medical University, Guangzhou, Guangdong, China

**Keywords:** extracellular matrix, kidney organoids, decellularized extracellular matrix, microenvironment, disease models

## Abstract

The kidney plays a crucial role in maintaining the body’s microenvironment homeostasis. However, current treatment options and therapeutic agents for chronic kidney disease (CKD) are limited. Fortunately, the advent of kidney organoids has introduced a novel *in vitro* model for studying kidney diseases and drug screening. Despite significant efforts has been leveraged to mimic the spatial-temporal dynamics of fetal renal development in various types of kidney organoids, there is still a discrepancy in cell types and maturity compared to native kidney tissue. The extracellular matrix (ECM) plays a crucial role in regulating cellular signaling, which ultimately affects cell fate decision. As a result, ECM can refine the microenvironment of organoids, promoting their efficient differentiation and maturation. This review examines the existing techniques for culturing kidney organoids, evaluates the strengths and weaknesses of various types of kidney organoids, and assesses the advancements and limitations associated with the utilization of the ECM in kidney organoid culture. Additionally, it presents a discussion on constructing specific physiological and pathological microenvironments using decellularized extracellular matrix during certain developmental stages or disease occurrences, aiding the development of kidney organoids and disease models.

## 1 Introduction

The kidney, is a vital organ that plays an important role in maintaining the homeostasis of human body. Chronic kidney disease (CKD), a progressive and irreversible loss of kidney function, is becoming increasingly prevalent due to rising comorbidities such as diabetes, hypertension, obesity, and an aging population (Kishi et al., 2024; GBD Chronic Kidney Disease Collaboration, 2020). Current therapeutic approaches for CKD are limited, relying predominantly on antihypertensive agents, antidiabetic medications, and pharmacological strategies aimed at controlling disease progression (Kishi et al., 2024). These treatments, required prolonged administration and exhibited only moderate efficacy, failing to halt the progression of kidney injury to end-stage kidney disease (ESKD), defined as an eGFR below 15 mL/min/1.73 m^2^ (Levey et al., 2005). Animal disease models has substantially enhanced our understanding of the CKD pathophysiology and the clinical pharmacodynamics (Schnell et al., 2022; Fu et al., 2024). However, the interspecies differences significantly hinder the accurate extrapolation of disease mechanisms and the therapeutic efficacy. This challenge emphasizes the necessity for improved disease models that accurately reflect renal pathogenesis and facilitate precise drug screening approaches (Musah et al., 2024).

Organoids, as self-assembled 3D cellular structures *in vitro*, retain key characteristics of their *in vivo* counterparts and have emerged as powerful tools for developmental biology and drug screening. Kidney organoids have been a subject of research for nearly a decade (Taguchi et al., 2014). Over this time, their maturation has steadily advanced, enabling their use in constructing kidney disease models and drug screening (Musah et al., 2024; Tabibzadeh and Morizane, 2024; Dilmen et al., 2024; Long et al., 2024; Oishi et al., 2024; Chambers et al., 2023). However, their functional maturation and structural organization remain a challenge, in part due to the complexity of the kidney microenvironment (Garreta et al., 2019). The integration of extracellular matrix (ECM) components into organoid cultures has emerged as a promising strategy to mimic the *in vivo* environment, supporting more accurate tissue development and improving the functionality of kidney organoids (Kim J. W et al., 2022; Lacueva-Aparicio et al., 2022).

**FIGURE 1:**
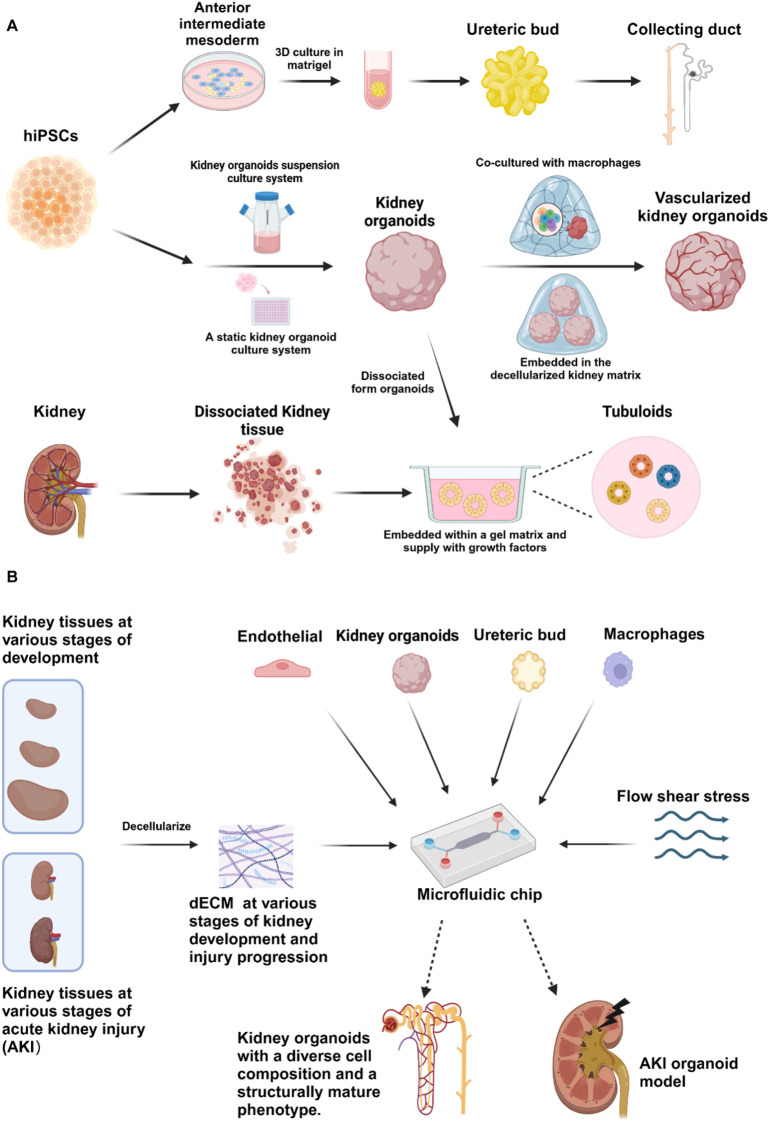
Various strategies are employed in the fabrication of kidney organoids (Created in BioRender.com). **(A)** Current procedures employed to construct ureteric bud (UB) organoids, kidney organoids and tubuloids using iPSCs and primary cells obtained from renal tissues. UB organoids are developed by differentiating hiPSCs into anterior intermediate mesoderm, which is subsequently embedded in Matrigel for 3D culture, leading to the formation of UB organoids that further differentiate into organoids containing collecting ducts. Kidney organoids, on the other hand, can be generated from hiPSCs using either suspension or static culture systems, with variations in the cultivation process resulting in vascularized kidney organoids. Finally, tubuloids are derived by embedding cells within a gel matrix and supplementing them with growth factors; their cellular origin may be either primary renal cells or dissociated organoids. Solid arrows indicate the processing steps, while dashed lines signify areas that have been magnified for clarity. **(B)** Schematic representation of further advancements in generation of sophisticated kidney organoids and acute kidney injury (AKI) organoid model achieved on organoids-on-chip system, incorporating the induction of decellularized renal extracellular matrix from specific developmental and pathological stages, co-culture with various exogenous cells, and introduction with fluidic shear stress, in order to simulate *in vivo* microenvironment. Solid arrows indicate the processing steps, while dashed arrows represent abstract model diagrams.

This article provides a comprehensive review of the current methods for the cultivation of kidney organoids (Figure 1A), with a particular focus on discussing the advantages and disadvantages of utilizing ECM for the culture of kidney organoids. It also proposes corresponding improvement strategies and outlines the future directions for the cultivation and application of kidney organoids.

## 2 Strategies for the construction of kidney organoids

Based on the single-cell sequencing data, 25 distinct cell types have been identified within the adult kidney tissue (Balzer et al., 2022). The development of the mammalian kidney initiates with the emergence of the nephrogenic cord, which is sequentially exposed to Wnt/β-catenin and BMP signaling to form the intermediate mesoderm (IM) (Schnell et al., 2022). This process provides two sources of progenitor cells for the differentiation of the collecting duct (CD) and the functional kidney units. Specifically, it involves the ureteric bud (UB), which originates from the anterior intermediate mesoderm (aIM), and the metanephric mesenchyme, which arises from the posterior intermediate mesoderm (pIM). However, currently kidney organoid culture strategies are unable to simultaneously provide appropriate culture conditions for both types of progenitor cells, which are requisite for replicating the reciprocal inductive signals observed *in vivo*. Specifically, GDNF secreted by MM initiates UB branching, while WNT9B secreted by UB initiates the mesenchymal-to-epithelial transition of the nephron (Oxburgh, 2018). Since the first report of nephron organoids induced from hiPSCs in 2014 (Taguchi et al., 2014), various protocols for constructing nephron organoids have endeavored to mimic the early kidney embryonic development. These protocols involved the induction of mesoderm formation in embryoid bodies through the activation of BMP4 and WNT signaling pathway using the GSK3 inhibitor (CHIR99021) (Lindsley et al., 2006; Magro-Lopez et al., 2024), followed by exposure to FGF9 to induce and maintain the niche of nephron progenitor cells (NPCs) (Muthukrishnan et al., 2015). The advancements in these methods have enabled the development of kidney organoids that provide a model that closely resembles human physiology, allowing the study of kidney biology at the organ level, and is superior to traditional two-dimensional culture systems or non-primate models.

Over the past decade, several laboratories have consistently improved the process of lineage reproduction of kidney organoids *in vitro*. Resulting in models that feature a broader spectrum of specialized cell types and increased structural complexity (Table 1). These advancements have proven to be crucial in understanding the pathogenesis of human kidney diseases and facilitating extensive drug screening (Taguchi and Nishinakamura, 2017; Takasato et al., 2014; Takasato et al., 2015; Kumar et al., 2019; Freedman et al., 2015; Huang et al., 2024; Li et al., 2016; Low et al., 2019; Morizane and Bonventre, 2017; Lawlor et al., 2021) (Table 1). However, the current established kidney organoids are characterized by an immature fetal state and transcriptionally similar to the first or second trimester of human fetal kidney, and lack an integrated vascular system, severely limiting their growth rate and long-term culture *in vitro*. A multimodal atlas of kidney organoid differentiation has delineated at least 15 highly specialized cell types, with off-target cell proportions varying from 6% (Combes et al., 2019) to 20% (Wu et al., 2018). Notably, the reproduction of distal cell types (mainly distal tubule and collecting duct cells) in organoids is comparatively less sophisticated than that of proximal cell types (mainly proximal tubule cells) (Yoshimura et al., 2023). Consequently, although kidney organoids demonstrate morphological similarity to the developing renal tissue, they encounter significant hurdles in attaining complete maturation and intricacy, especially with regard to replicating the *in vivo* filtration capabilities.

**TABLE 1:** Induction strategies for kidney organoids.

Ref.	Sources	Improvement	Method	Organoids
Krupa et al., 2024 (PMID: 38984433)	hiPSCs	Support the growth and maturation of kidney organoids	Cultured in Self-assembling polypeptide hydrogels and GelMA hydrogels	Kidney organoids
Garreta et al. (2019) (PMID:30778227)	hiPSCs	Accelerate the differentiation of kidney organoids	Cultured in 1kp polyacrylamide hydrogel	
Homan et al., 2019 (PMID:30742039)	hiPSCs	Generates vascularized kidney organoids with more mature podocyte, enhanced cellular polarity	3D-printed millifluidic chips were embedded in gelatin-fibrin ECM and applied for low or high fluid shear stress	
Sun et al. (2020) (PMID:32698872)	hiPSCs	The usc organoids self-organized well with no significant cell death	Cultured in optimal kidney ECM	Usc organoids
Lee et al., 2021 (PMID:34748091)	hiPSCs	More mature podocytes and vascular structures	Microfluidic chip was coated with 1.5% Matrigel and 1.5% Matrigel containing 100 ng/mL VEGF	Kidney organoids
Kim J. W et al. (2022) (PMID:35322595)	hiPSCs	Increase the formation of blood vessel network and promote the maturation of kidney organoids	Kidney decellularized matrix hydrogel	
Nerger et al., 2024 (PMID:38180232)	hiPSCs	Affect the roundness of nephron segments, spatial localization and the ratio of glomerulus to tubules	Sodium alginate hydrogel	
Pecksen et al. (2024) (PMID:38702808)	hiPSCs	Decrease the apoptosis of iPSCs induced by CHIR, promote the differentiation of renal organoids and promote vascularization	A monocyte of human origin coculture with iPSCs by transwell cuture system	
Garreta et al. (2024) (PMID:38762768)	hiPSCs	Maintain organoid differentiation and promote vascularization	Kidney derived decellularized matrix hydrogel, hPSCs derived endothelial organoids were embedded for three-dimensional culture	
Maggiore et al. (2024) (PMID:38901605)	hiPSCs	Increase the vascularization of renal organoids and improve the maturity of nephron. Generate different endothelial cell subtypes	iETV2-iPSCs were integrated into the previously constructed renal organoid system, and ETV2 expression was induced at day5 after culture	
Takasato et al. (2015) (PMID:26444236)	hiPSCs	The first report for *in vitro* kidney organoids induction; Optimization the exposure period of Wnt, FGF, and RA in fate selection	2D induced cell were spun down to form a pellet and transferred onto a Transwell under the sequential induction by Wnt, FGF	
Freedman et al. (2015) (PMID:26493500)	hiPSCs	Simplified induction procedure; validate the Genome-modified kidney organoids form PKD-specific cysts	Induction Procedure followed by the formation of cavitated spheroids, then through MET induction to form nephron-like organoids	
Morizane et al. (2015) (PMID: 26458176)	hiPSCs	First reported strategy to replated 2D NPCs into 3D suspension culture with distinct lumens mimics the nephron	Generation of SIX2+SALL1+WT1+PAX2+ NPCs with high efficiency followed by the formation of PAX8+LHX1+ renal vesicles that self-organized into 3D nephron structures	
Taguchi and Nishinakamura (2017) (PMID:29129523)	Mouse and hiPSCs	Higher-order structures with branching morphogenesis by co-culturing independently induced ureteric bud, nephron and stromal progenitor lineages	Identified mutually distinct inductive signals between the NP and UB lineages in every step of differentiation; Co-culture iNPs, iUB, and the Pdgfra+ SP population sorted from E11.5 embryonic kidneys. iUB: induced UB; iNPs: NP induction from the mESCs; SP: stromal progenitors	
Li et al. (2016), Huang et al. (2024) (PMID:27570066 PMID:38692273)	Primary mouse and human NPCs/hiPSCs	Develop systems for long-term expansion of induced NPCs generated nephron organoids with minimal off-target cell types and enhanced maturation of podocytes relative to other strategies; podocyte reprogramming to an NPC-like state	Manipulation of p38 and YAP activity allowed for long-term clonal expansion of primary mouse and human NPCs and induced NPCs from hiPSCs	
Vanslambrouck et al., 2022, 2023 (PMID:37770563 PMID:36209212)	hiPSCs	PT-enhanced organoids with distinct S1 - S3 proximal tubule cell types; improved albumin and organic cation uptake, improved expression of SARS-CoV-2 entry factors resulting in increased viral replication	Extended the monolayer differentiation of nephron progenitor to 12–14 days cultured in enhanced BMP7 condition to 10 ng/mL	
Przepiorski et al. (2018), Przepiorski et al. (2021) (PMID:30033089 PMID:33938892)	hiPSCs	Establish a fast, efficient and cost-effective suspension culture method, allows large-scale organoid production	Bioreactor-Based	
Kumar et al. (2019) (PMID:30846463)	hiPSCs	Modified suspension culture method, a three- to fourfold increase in final cell yield and a 75% reduction in cost per million organoid-derived kidney cells compared with static culture	Low speed (60 rpm) swirling on an orbital shaker to form cell aggregates	
Lawlor et al. (2021) (PMID:33230326)	hiPSCs	Rapid and high-throughput; highly uniformly patterned; increasing nephron yield	3D bioprinting	
Howden et al. (2021) (PMID:33378647)	Distal nephron epithelim from kidney organoids	Shift the identity of this GATA3+/EPCAM + epithelial population toward UE, including ureteric tips, cortical and medullary UE, by altering the *in vitro* culture conditions	GATA3+/EPCAM + epithelial population isolated by FASC and induced in the presence of GDNF, CHIR, FGF2, ATRA and Y-27632	UB/Collecting duct organoids
Mae et al. (2020) (PMID:32726627)	hiPSCs	Induced UB organoids have tubular lumens and repeat branching and differentiated into collecting duct progenitors.	iPSC induced to AIM and then ND stage embedded in 2% Matrigel for 6 days to constitute induced UB organoids with epithelial polarity and tubular lumens	
Uchimura et al. (2020) (PMID:33326782)	hiPSCs	Combine independently differentiated MM-like and UB-like progenitors to generate human kidney organoids with a collecting system. Detect for the first time urothelial (Uro) cells	Aldosterone and AVP drive collecting duct maturation	
Zeng et al. (2021) (PMID:34131121)	Mouse and human fetal kidneys; hiPSCs	UB organoids generate collecting duct organoids, with differentiated principal and intercalated cells Develope a screen to establish conditions supporting the differentiation of CD organoids	Sorting of KIT + cells were used to enrich the precursor population then induced in chemically-defined culture conditions	
Shi et al., 2023 (PMID:36038632)	hiPSCs	Exhibit authentic morphological behavior and responses to developmental stimuli; recapitulate the morphogenetic pattern in isolated UBs	Ensure at least 90% efficiency at the mesendodermal and pronephric intermediate mesoderm (IM) stages without using cell sorting or mechanical dissection	
Yousef Yengej et al. (2023) (PMID:36724260)	iPSC-derived organoids	Selectively expand the mature functional renal epithelium without off-target cells and provide easy apical access that enables evaluation of tubular transport	Tubular fragments and cells from D7+18 organoids resuspended in Basement Membrane Extract (BME) gel and plated on suspension culture wells plates	Tubuloid organoids
Lindoso et al., 2022 (PMID:35841001)	Tubuloid-derived cells + EVs	EVs from kidney tubular epithelial cells can phenotypically improve *in vitro* tubuloid maturation	Tubuloids cultured with EV	
Schutgens et al. (2019), Gijzen et al. (2021) (PMID:30833775 PMID:33674788)	Adult kidney tissue or urine	Long-term growth and can be expanded for at least 20 passages; Model infectious, malignant and hereditary kidney diseases; Adopt a tubular conformation and display active (trans-) epithelial transport function	Establishing kidney tubuloids and characterization of tubuloid cell–derived 3D tubular structures in a perfused microfluidic multi-chip platform, the OrganoPlate 3-Lane	

Ref. for Reference, hiPSCs, for human indued pluripotent stem cells; USCs, for urine-derived stem cells, Extracellular vesicle (EVs), Ureteric bud (UB), Nephron progenitor cells (NPCs).

Furthermore, attempts have been dedicated to construct higher-order kidney organoids with higher lineage integrity and fully recapitulating *in vivo* renal developmental structures. To achieve this, some groups have developed ureteric bud (UB)/CD organoids derived from hiPSCs or UB progenitor cells extracted from mouse and human fetal kidneys, characterized by expandable, serially passaged and repeat branching morphogenesis (Howden et al., 2021; Mae et al., 2020; Shi et al., 2023; Uchimura et al., 2020; Zeng et al., 2021). Additionally, several proof-of-concept studies generated engineered kidney by aggregating 3D co-cultured NPCs with UB organoids derived from mice (Zeng et al., 2021), which preliminarily replicated the interconnected nephron and CD structures mimicking the reiterative inductive process of kidney development *in vitro*, shedding light on the developmental and regeneration mechanisms of the CD system (Zeng et al., 2021). However, akin to the limitations of nephron organoids, CD organoids derived from UB progenitors remain phenotypically immature compared to their *in vivo* counterparts, and functional evaluation demonstrating secretion and electrolyte reabsorption process is yet to be fully established (Mae et al., 2020).

To overcome the limitations of the extensive induction time and inadequate maturity of kidney organoids, researchers have turned to the induction and cultivation of tubuloids derived from primary renal tubular epithelial cells which were also found in urine (Gijzen et al., 2021; Schutgens et al., 2019). Intriguingly, tubuloid-derived cells can form polarized, leak-tight kidney tubules capable of performing trans-epithelial transporter activity (Yousef Yengej et al., 2023). Tubuloids serve as a highly physiologically relevant model for simulating infectious, malignant, and genetic kidney diseases, including tubulopathies such as Fanconi syndrome (Jamalpoor et al., 2021), re-invigorating the understanding of renal transport mechanisms, drug screening, and personalized medicine. Nonetheless, the capacity of tubuloids to accurately replicate diseases with complex multi-cellular interactions and intricate pathological mechanisms necessitates further investigation.

Although kidney organoid with similar degrees of differentiation have been established via cohort of procedures, most induction programs are time intensive and require the administration of costly exogenous growth factors, severely limiting the development of large-scale organoid culture strategies (Morizane and Bonventre, 2017). The complexity and high costs associated with these methods pose significant barriers to their widespread use in research and therapeutic applications. Therefore, establishing a controllable, and highly reproducible culture process, is essential for optimizing the lifespan, architecture complexity, homogeneity, and differentiation fidelity of organoids. This prospect is crucial for the standardization and large-scale generation of the next-generation organoids. Alan’s (Przepiorski et al., 2018; Przepiorski et al., 2021) and Little’s laboratory (Kumar et al., 2019) have developed suspension organoid culture systems resulted in a 3-4 fold increase in final cell yield compared to static culture, providing a highly promising platform for the automation and large-scale production of kidney organoids. Furthermore, the integration of bioprinting technologies can automate the production of organoids with highly homogeneity in conformation, improving the throughput of manufacture up to 9 fold (Lawlor et al., 2021). The large-scale production of kidney organoids via bioengineering strategies provides versatile platform for optimizing organoid technology towards revolutionizing the regenerative medicine and clinical applications. The integration of automated high-throughput imaging techniques enables the phenotypic analysis of kidney organoids, which can be utilized for drug screening related to nephrological disorders (Czerniecki et al., 2018; Wang et al., 2022).

In summary, the advancements of kidney organoids with the capability of recapitulating early temporal-spatial embryonic developmental trajectories are now used as faithful substitutes in studying kidney development *in vitro*. Organoid models enable the reproduction of tissue structures, providing opportunities to investigate the mechanism of kidney development and disease through functional screening (Huang et al., 2024). However, the efficacy of kidney organoid models depends on their developmental fidelity to primary tissue, and to what extent can they mimic embryonic organ development on cellular characteristics and architectural complexity levels remains challenging (Little and Combes, 2019). Consequently, modifying the *in vivo* biophysical microenvironment in spatiotemporal dimensions exhibits great potential for driving the determination of cell fate and commitment to lineage during organoid development.

## 3 The construction of a microenvironment specific to kidney tissue facilitates the differentiation of kidney organoids

Early embryonic development involves the formation of three germ layers, where cell fate is regulated by intracellular and extracellular signaling pathways. A multitude of studies have underscored the determinant role of specific transcription factors in lineage commitment (Wang et al., 2013; Takahashi and Yamanaka, 2006; Rigillo et al., 2021; Chen et al., 2024). However, research on how to specifically regulate cell fate through extrinsic signals is still limited (Walma and Yamada, 2020). The ECM significantly influences cell fate by activating various signaling pathways, which play a crucial role in cell fate decisions (Tang et al., 2022; Tang et al., 2013; Amran et al., 2024; Kersey et al., 2024; Li et al., 2024; Zhang et al., 2023; Sun et al., 2020). Therefore, elucidating the composition and dynamic changes of the ECM during the processes of cell development, aging, and disease progression is crucial for simulating and constructing the microenvironment of tissue at different developmental stages, injuries, and pathological processes.

The complexity of the ECM arises from its diverse constituents, including core structural proteins and regulatory factors that can initiate ECM remodeling and impact development and disease (Yamada et al., 2022; Rekad et al., 2022; Zhou et al., 2018; Damjanovski et al., 2001; Kaneko et al., 2024). Advances in tissue engineering allow for the simulation of ECM using biomaterials to achieve *in vitro*/*in vivo* cell fate regulation. However, disparities exist between commercial biomaterials and tissue ECM, which influence cell fate regulation and the efficacy of disease treatment (Zhang et al., 2023; Kim S. et al., 2022). Decellularized extracellular matrix (dECM) hydrogels are prepared through chemical or physical decellularization processes that remove immunogenic and pathogenic elements from natural tissues, followed by freeze-drying, grinding, and enzymatic digestion. These hydrogels retain the majority of bioactive proteins from the original tissue (Zhang W. et al., 2021). Therefore, the use of dECM derived from tissues to simulate the physiological microenvironment has attracted increasing attention for organoid studies. For example, Sun et al. demonstrated that the dECM hydrogels from spinal cord of neonatal rabbits can promote the axonal growth and functional maturation of spinal cord organoids (Sun et al., 2024). Similarly, in the aging process, the composition and mechanical properties of the ECM have also changed, thereby affecting tissue function. Culturing normal human mammary epithelial cells with the ECM from aged breast tissue reinforced the invasive capability of cells, and increased the expression of inflammatory cytokines and cancer-related genes and proteins (Bahcecioglu et al., 2021). Moreover, cervical squamous cell carcinoma (CSCC) patients’ adjacent cervical tissue can be used to prepare uterine cervical extracellular matrix (UCEM) hydrogels, which faithfully defined the microenvironment of cervical cancer tissue. CSCC organoids cultured with UCEM hydrogel exhibit superior characteristics compared to those cultured with Matrigel, as evidenced by increased expression of cervical cancer-related genes and signaling pathways, resulting in a closer resemblance to patient-derived CSCC tissues (Song et al., 2024). The above studies indicated that the preparation of dECM from tissues under different physiological/pathological conditions can help construct more mature organoids and disease models.

The influence of extracellular matrices (ECMs) on renal development and functionality has been extensively investigated, yielding insights into various aspects such as kidney morphogenesis, branching patterns, pathologies, and regenerative processes (Abdollahzadeh et al., 2022). Several research groups have employed proteomics to analyze the ECM composition in normally developing kidneys, aging kidneys, and kidney diseases (Diedrich et al., 2024; Rende et al., 2023; Randles et al., 2021; Eckersley et al., 2023; Li et al., 2023; Lipp et al., 2021; Lennon et al., 2014). Understanding the composition and dynamic changes of kidney ECM under different physiological and pathological conditions provides the basis for constructing microenvironments of renal tissues with diverse physiological and pathological characteristics. Furthermore, a series of studies has utilized kidney dECM for renal cell culture (Quinteira et al., 2024; Bongolan et al., 2022; Sobreiro-Almeida et al., 2020), renal injury repair (Kim et al., 2024), and organoid culture (Kim J. W et al., 2022; Garreta et al., 2024). In terms of renal cell culture, dECM-based hydrogels have been shown to effectively support renal progenitor cell survival, proliferation, and differentiation into tubular cells and podocytes, thereby providing a biocompatible platform conducive to renal regeneration (Quinteira et al., 2024). Furthermore, optimizing the decellularization process—such as using lower concentrations of SDS during the procedure—helps to preserve essential ECM components, enhancing renal cell survival and distribution, although challenges remain regarding mature cell migration (Bongolan et al., 2022). Additionally, dECM can serve as a substitute for the tubular basement membrane, simulating the physiological relevance of the *in vivo* environment. Co-culturing renal progenitors with endothelial cells has enabled the construction of a tubular bilayer model, which mimics the native tissue environment more closely (Sobreiro-Almeida et al., 2020). In the aspect of renal injury repair, an implantable decellularized extracellular matrix sponge has demonstrated not only rapid hemostasis during partial nephrectomy surgery but also superior wound healing, offering a promising solution for both managing renal hemorrhage and enhancing tissue regeneration at the lesion site (Kim et al., 2024). Collectively, these studies highlight the potential of dECM to advance renal research and therapeutic applications, including the enhancement of renal cell cultures and injury repair. Decellularized materials created in various laboratories have demonstrated the ability to promote differentiation, maturation, vascularization, and the development of tubular and glomerular-like structures in kidney organoids (Kim J. W et al., 2022; Garreta et al., 2024), reinforcing the promising role of dECM in advancing both basic research and clinical applications. Additionally, some laboratories have developed decellularized matrices from fibrotic kidneys to assess the impact of dECM on endothelial progenitor cells (Zhang R. et al., 2021). Although studies have not yet reported how these dECMs derived from pathological kidneys impact kidney organoid differentiation, they hold potential for constructing disease model organoids that may better simulate pathological conditions. However, there are still gaps in the maturity of kidney organoids (including the presence of precursor cells and cell cycle cells), the representation of cell types (lacking pericytes and distal tubular cells), and structural complexity (vascular wrapping and podocyte wrapping structures) compared to mature renal tissues (Kim J. W et al., 2022). In addition, the kidney organoids may contain off-target cell populations (Kim J. W et al., 2022). One potential explanation is that the current manufactured dECM primarily recapitulates the matrue renal-favor microenvironment. In contrast, kidney organoids are usually generated from hiPSCs, which contain numerous cells in the early stages of differentiation. As a result, the dECM derived from mature tissues may not be optimal for supporting the maturation of these early-stage differentiated cells in kidney organoids, leading to hindrances in their development. This mismatch between dECM derived from mature tissue and kidney organoids composed of early-stage differentiated cells highlights a crucial challenge in the field. Despite the absence of direct studies comparing early-stage and mature kidney dECM in renal organoid cultures, clues can be drawn from existing studies on the dECM in other organ systems. For instance, a study on rabbit spinal cord dECM found that neonatal dECM contained higher levels of proteins like pleiotrophin (PTN) and tenascin (TNC), which promote neural development, axonal growth, and regeneration, while mature dECM had more inhibitory components like chondroitin sulfate proteoglycans (CSPGs), limiting regenerative potential (Sun et al., 2024). This shift in ECM composition highlights a potential mismatch when applying mature tissue-derived ECM to support the maturation of progenitor cells in organoids. Early-stage ECM is optimized for promoting cell proliferation and differentiation, while mature ECM may lack these developmental cues, potentially hindering organoid maturation and limiting its functionality. By understanding and mimicking the developmental ECM environment, researchers may be able to better support the maturation and functional development of organoids, leading to more effective tissue models for both research and therapeutic applications.

## 4 Conclusion and prospect

Amidst the rapid advancements in multidisciplinary technologies, despite significant advancements in cellular diversity, structural complexity, functional repertoire, and developmental maturity of kidney organoids, a discernible disparity remains when compared to mature renal tissues. To address this, one potential method is the construction of a tissue microenvironment based on tissue-specific dECM, which could facilitate the maturation of kidney organoids. Both human and porcine renal dECM have been found to promote the differentiation of kidney organoids (Garreta et al., 2024). This discovery not only paves the way for potential commercialization of renal dECM but also addresses ethical concerns related to the use of human dECM.

Although current dECM derived from mature renal tissues can partially promote the maturation and vascularization of organoids, there are limitations in terms of cell types and structures, with the presence of non-renal cell types. The continued differentiation and maturation of kidney organoids require an ECM that is distinct from mature renal tissues. To address this, single-cell sequencing can be utilized to analyze various stages of kidney development and aging, as well as different regions. Furthermore, the ECM can be identified using mass spectrometry. Through the integration and comparison of single-cell multi-omics data at different development stages of kidney and kidney organoid differentiation, it becomes possible to identify the ECM that best corresponds to the kidney organoids. Culturing organoids with the corresponding stage’s ECM and introducing exogenous cells such as macrophages (Liu et al., 2020; Pecksen et al., 2024) and endothelial cells (Maggiore et al., 2024), a complex cellular microenvironment can be constructed to simulate physiological conditions to the greatest extent (Figure 1B). Furthermore, microfluidic chips can be utilized to apply fluid shear stress to the three-dimensional co-cultured organoids, thereby mimicking the processes of kidney development, aging, and disease (Figure 1B). Developing organoids at these specific stages can help elucidate the mechanisms of development, aging, and disease occurrence, and also provide a promising direction for drug screening in nephropathy using kidney organoids.

There are numerous causes of kidney disease, including congenital genetic conditions such as polycystic kidney disease (Cornec-Le Gall et al., 2018), as well as a significant proportion of kidney diseases induced by nongenetic factors, such as obstructive nephropathy or nephrotoxic drugs leading to acute kidney injury (AKI) (Chávez-Iñiguez et al., 2020; Perazella and Rosner, 2022). Genetic factors, which induced kidney diseases can be modeled by gene editing of hiPSCs followed by the induction of kidney organoid to obtain the corresponding disease models. However, there is still limited research on how to construct kidney organoid disease models induced by nongenetic factors. Although several organoids models of AKI have been developed through the use of various inflammatory stimuli or nephrotoxic drugs (Morizane et al., 2015), there is still a certain gap between these organoid models and AKI due to the maturity of organoids (Bejoy et al., 2022). Moreover, due to the multitude of causes of AKI, various alterations in ECM proteins are also markers of AKI, such as nidogen-1 glycoprotein (Gui et al., 2024) and Metalloproteinase 1 and 3 (Klimm et al., 2024). However, our understanding of the dynamics of the overall ECM composition and cellular microenvironment during the occurrence and development of AKI is still limited. Therefore, how to use the ECM related to AKI diseases in combination with kidney organoids to construct a more physiologically relevant AKI model is also a direction for future research.

In summary, kidney organoids serve as crucial multicellular models for studying renal development, aging, and disease *in vitro*, and offer distinct advantages over traditional animal and cell models. Their greatest strength lies in the presence of multiple interacting cell types and a certain level of physiological structure, allowing them to simulate the microenvironment of kidney tissue *in vitro*. However, there remains a gap between current kidney organoids and mature renal tissues, both in terms of cell types and maturity. Furthermore, there is limited research on constructing organoids that precisely mimic specific stages of human kidney development, aging, and disease. One viable approach to address these challenges involves utilizing kidney dECM that correspond to the developmental stages of tissue. By co-culturing immune-related cells and creating a complex cellular microenvironment that closely resembles physiological conditions, it becomes possible to obtain more differentiated cell types and maturity in kidney organoids. Subsequently, these advanced models enable more accurate and reliable drug screening. Furthermore, the application of microfluidic chip technology enables the construction of micro-physiological models that replicate multi-organ interactions in disease states, facilitating the study of organ interactions under normal physiological and disease conditions and also drug screening. These avenues represent future directions for the advancement of kidney organoid research.
